# Advances in Optical Sensing Technologies for On-Site Detection of Harmful Residues in Food: Principles and Recent Applications

**DOI:** 10.3390/foods14234073

**Published:** 2025-11-27

**Authors:** Qinghua Liu, Yuanyuan Liu, Huihui Yang, Qian Su, Linglei Li, Xiangqi Meng, Minmin Li, Xiaoxue Jia, Peihua Ma, Bei Fan, Fengzhong Wang, Long Li

**Affiliations:** 1 Institute of Food Science and Technology, Chinese Academy of Agricultural Sciences, Beijing 100193, China; 15104286403@163.com (Q.L.); yanghh_6@163.com (H.Y.); lll8880223@163.com (L.L.); xm93@cornell.edu (X.M.); liminmin@caas.cn (M.L.); jiaxiaoxue@caas.cn (X.J.); ma.peihua@outlook.com (P.M.); fanbei5172021@126.com (B.F.); wangfengzhong@sina.com (F.W.); 2 College of Mechanical and Electrical Engineering, Tarim University, Alar 843300, China; 3 Modern Agricultural Engineering Key Laboratory at Universities of Education Department of Xinjiang Uygur Autonomous Region, Tarim University, Alar 843300, China; 4 XXPCC Key Laboratory of Utilization & Equipment of Special Agricultural and Forestry Products in Southern Xinjiang, Tarim University, Alar 843300, China; 5 Weifang Institute of Food Science and Processing Technology, Weifang 261000, China; suqian1256@126.com; 6 CAAS East Center (Suzhou) for Agricultural Science and Technology, Suzhou 215000, China

**Keywords:** optical sensing technologies, on-site detection, food testing, food safety, fluorescence imaging, ultraviolet–visible absorption spectroscopy, surface-enhanced raman spectroscopy, colorimetric method

## Abstract

Food safety has recently attracted increasing attention, underscoring the need for timely and accurate on-site testing technologies. Optical detection, among various methods, offers notable advantages, including ease of use and rapid results, making it a promising approach for food safety applications. This paper reviews the fundamental principles of optical inspection for food field examination and explores its practical applications, including techniques such as surface-enhanced Raman scattering, UV–visible absorption spectroscopy, and fluorescence detection. Furthermore, this review discusses the integration of detection technologies with nanotechnology and smartphone-based systems. In addition, this review discusses the current applications, challenges, and potential solutions associated with optical detection in on-site food inspections.

## 1. Introduction

**Figure 1:**
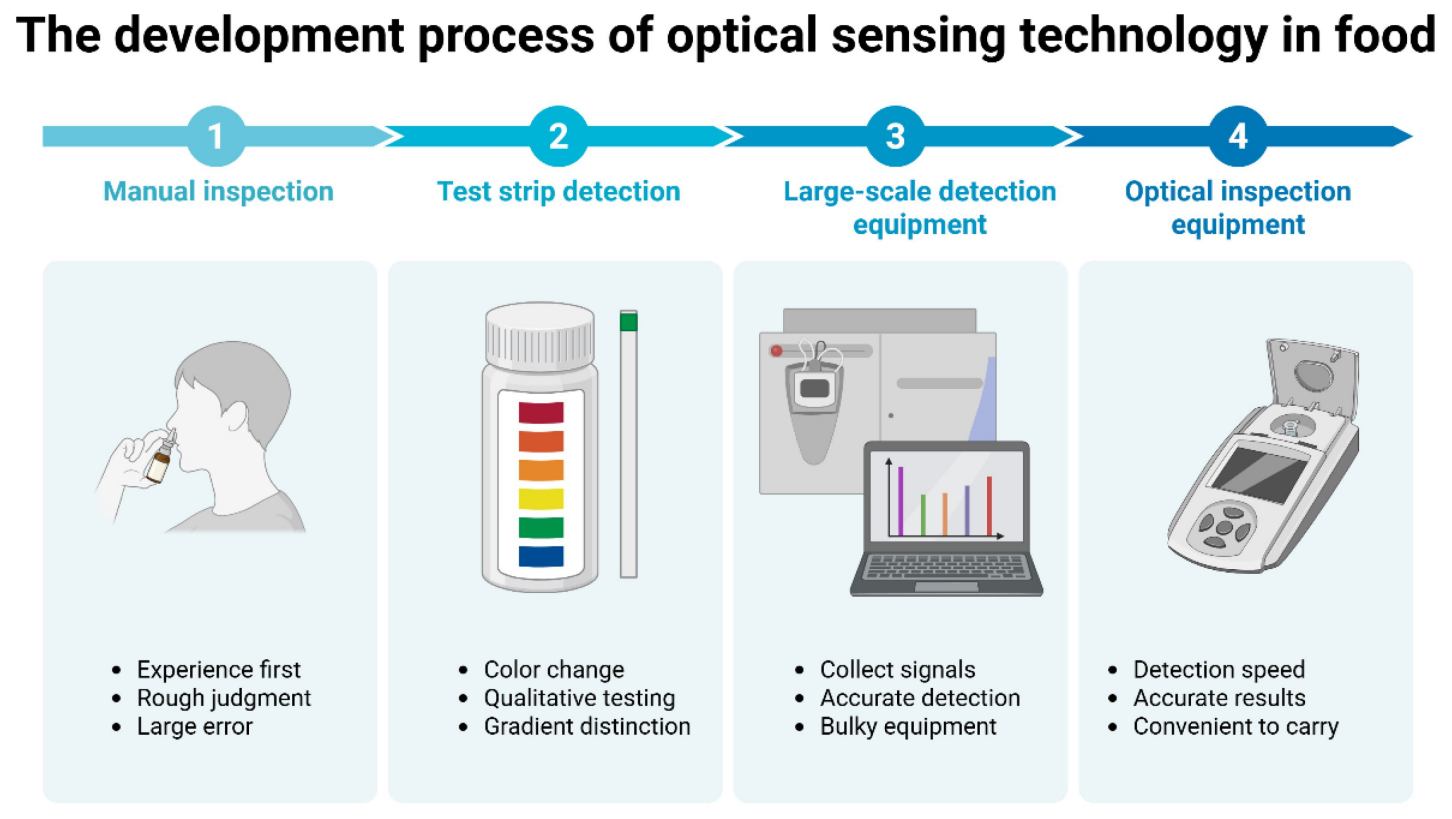
Roadmap of development progress in optical sensing technologies for on-site detection of harmful residues in food.

Adequate nutrition is a fundamental prerequisite for human health. Stringent standards have been implemented for food quality and safety to enhance supervision. According to statistics from the World Health Organization (WHO), approximately 600 million people worldwide suffer from foodborne diseases each year, of whom 420,000 die [1]. Meanwhile, food adulteration causes economic losses of up to 40 billion US dollars annually [2]. Food safety and quality are affected by a series of hazards, which continuously penetrate food matrices through cumulative bioaccumulation pathways and permeate all key nodes of the food supply chain [3]. According to the Food Safety Law of the People’s Republic of China, there are 1070 national food standards and 1164 industry standards in place. Consequently, new requirements have been established to support food testing technologies, including specificity, non-destructive analysis, rapid detection, and accuracy [4]. Currently, the most prevalent detection methods are chemical analysis and biological cultivation. These detection methods not only impose high requirements on personnel and instrumentation but also entail significant costs and extended detection periods. Furthermore, the signal in food detection may be weak when the addition of certain illegal substances is minimal. However, even small quantities may pose a threat to human health [5]. Therefore, there is a growing demand for sensitive, simplified, and rapid on-site food detection equipment for law enforcement, food sampling, and routine monitoring processes. Figure 1 illustrates the development progress of on-site detection technologies for harmful residues in food.

Various chemical and biological methods have been developed and applied for food detection, including bacterial culture counting, polymerase chain reaction (PCR), high-performance liquid chromatography (HPC) [6], and capillary electrophoresis [7]. These technologies demonstrate high accuracy and low detection thresholds; however, the detection process requires extraction, which damages the tested material and results in significant sample loss. Furthermore, these methods require a series of processes, such as enrichment culture or purification analysis, to produce results, leading to extended analysis times. Additionally, certain detection techniques, such as PCR, are susceptible to interference from environmental factors or other substances in complex matrices, potentially yielding false positive results [8]. Therefore, there is a need for rapid detection methods in this field.

With the continuous advancement of optical detection technology, detection methods such as Raman spectroscopy, fluorescence detection, colorimetry, and UV–visible absorption spectroscopy (UV-VIS spectroscopy) have been applied for food detection [9], enabling rapid and non-destructive on-site detection [10]. Compared with traditional detection methods, optical detection methods offer advantages such as simplified detection steps, increased detection speed, and streamlined pretreatment processes, rendering them more suitable for on-site food detection requirements [11]. The application of optical detection combined with nanotechnology and smartphones may facilitate significant progress in the food field detection [12]. To meet the needs of on-site food inspection, optical inspection equipment is required to detect the objects under investigation with minimal or no damage while providing simple, rapid, and accurate results. In nanotechnology, metal nanoparticles and nanoenzymes can serve as probes [13] to enhance the signal of the test substance, specifically recognizing the test substance, and can be utilized for the development of food detection technology, reducing the size of detection instruments and achieving portable on-site detection [14]. Furthermore, the development of smartphones has led to intelligent applications for color analysis, which provide crucial support for on-site real-time detection through the analysis and optimization algorithm of spectral data combined with optical detection technologies, such as UV-VIS spectroscopy and colorimetry [15]. Significant progress has been made in the application of optical detection to food. Currently, food contamination and adulteration remain significant issues for human health and safety, and on-site optical detection technology is developing a potential for practical applications. The integration of emerging technologies, such as nanotechnology and optical inspection technology, has established a novel approach for non-destructive on-site inspection of food. Consequently, the on-site detection of food can identify and address the issues of food contamination and adulteration at a source.

Currently, several excellent literature reviews have discussed the detection of contaminants in food, focusing on the development of cutting-edge technologies [16,17] and the application of single technologies in on-site detection [18,19]. Thus, we integrate the advantages of multiple detection methods. This review focuses on the design, development, and recent advancements in various optical inspection methods suitable for on-site food inspection, which offer the advantages of being non-destructive, sensitive, accurate, and rapid. Currently, the optical detection methods reported to be applied in food field detection primarily include Raman spectroscopy, fluorescence spectroscopy, colorimetry, UV-VIS spectroscopy, and infrared spectroscopy. These optical detection methods predominantly rely on the development of novel nanoprobes or spectral data analysis to establish models and rapidly detect analytes by capturing and analyzing their signals. Therefore, we present a prospective outlook for optimizing optical sensors in combination with nanotechnology and mathematical models for use as a portable inspection method in the food field. This paper reviews the applications and challenges of optical detection technology in the field of food detection. This review provides novel insights into the on-site detection of harmful residues in food.

## 2. Principles of Optical Sensing Technology

### 2.1. Fluorescence Imaging

The fluorescence detection method involves irradiating the sample under investigation with excitation light of a specific intensity. Specific molecules or atoms in the sample or bound to it emit fluorescence, and the type and concentration of the substance are determined based on the wavelength and intensity of the emitted fluorescence [20]. Fluorescence detection offers ultra-low detection limits, high sensitivity, strong selectivity, and cost-effectiveness. When combined with nanotechnology, fluorescence detection overcomes the limitations of wide emission spectra and narrow excitation spectra, enabling the miniaturization of detection equipment and facilitating sensitive and efficient detection technology [21]. The development of high-performance aptamer sensors is crucial for ensuring an optimal fluorescence response from the target. Typically, fluorescent aptamer sensors consist of biomolecular aptamers coupled with signal transduction strategies [22].

Traditional fluorescent aptamer sensors generally operate on the “on-off” principle, wherein nanomaterials exhibit initial fluorescence. Upon introduction of the analyte, it interacts with the nanomaterial, resulting in fluorescence quenching. The degree of fluorescence quenching enables the detection of an analyte [23]. Specific fluorescent probes have been employed for various target analytes to ensure detection specificity [24]. In recent years, research has increasingly focused on the “on-off-on” mode of fluorescent aptamer sensors, which utilizes fluorescence resonance energy transfer (FRET), photoinduced electron transfer (PET), or the internal filtration effect (IFE) to quench nano-fluorescence and subsequently restore fluorescence upon addition of the target analyte [25]. This approach involves the measurement of the degree of fluorescence recovery of the target analyte. This method demonstrated enhanced detection accuracy and improved interference resistance [26].

Common fluorescent probe materials include nanomaterials, carbon dots, metal nanoclusters, and others. However, certain metal-based materials, such as rhodamine, are not only costly but also toxic, posing risks of chronic poisoning and even carcinogenicity [27]. This requires the development of environmentally friendly, eco-friendly, and safe alternatives. Currently, researchers are exploring inorganic, non-metallic fluorescent probes, such as carbon dots and semiconductor quantum dots. However, these materials often lack sufficient active sites on their surfaces, making them vulnerable to interference [28], which highlights the need for surface modification. Additionally, selectivity can be compromised in the presence of substances with similar properties. For instance, when detecting hydrogen sulfide (H_2_S), the presence of mercaptan analogs can significantly reduce the selectivity of the fluorescent probes [29]. To address these challenges, the development of probes with novel active sites is crucial. Consequently, achieving effective field applications for fluorescence detection requires the advancement of more selective probes and sensitive detection modes.

In recent years, natural nanoenzyme sensors have emerged as a significant research area owing to their high activity and good selectivity. However, the unclear catalytic activity of nanases may lead to inaccurate results. The combination of nanoenzymes and metal–organic framework, which possesses the characteristics of a large effective surface area and strong tunability, can functionalize nanoenzymes, improve accuracy, and enhance sensor sensitivity. Regarding fluorescent immune probes, Chen [30] incorporated La3+ ions into the original immune probe to detect zearalenone (ZEN) in corn. The addition of La3+ ions enhanced fluorescence performance. The fluorescence detection process exhibited increased efficiency and simplicity, aligning more closely with the requirements of grain field detection.

In the context of fluorescence luminescence mode, Li [31] modified the conventional “on-off” approach by utilizing the complementary aptamer sequence of ochratoxin A (OTA) DNA to effectively immobilize fluorescent silver nanoclusters. This modification resulted in a fluorescence quenching “off” state in the absence of OTA and a fluorescence “on” state in the presence of OTA. This model successfully mitigated the false positive effect caused by environmental interference, and the aptamer served as both a recognition agent and a quenching agent, eliminating the need for additional quenching agents or amplification processes. The sensor does not require expensive reagents, complex pretreatment, or sophisticated equipment to achieve the point-of-care detection of the target analyte.

### 2.2. Ultraviolet–Visible Absorption Spectroscopy

The UV-VIS spectroscopy absorption spectrum is generated by valence electron transitions, which typically refer to a series of spectra produced by the absorption of a subject under ultraviolet irradiation [32]. The energy levels corresponding to the molecules of different components vary, and consequently, the energy changes in the electronic transitions differ. Therefore, qualitative analysis can be conducted based on the shape and location of the absorption peak of the measured object. According to the Lambert–Beer law, when light is incident on a substance to be measured, the degree of absorption is proportional to its concentration, thus enabling quantitative analysis based on the intensity of the absorption peak [33].

The conventional UV-VIS spectroscopy absorption spectrometer has a complex structure consisting of a light source, monochromator, sample pool, detector, and output system [34]. Typically, the wavelength of the detected light ranges from 200 to 800 nm, and high-purity monochromatic light is generated using a monochromator. The absorption pool must be carefully selected and paired, as the absorption degree of the absorption pool itself also influences the UV-VIS spectroscopy absorption spectrum [35]. Traditional UV-VIS spectroscopy absorption spectrum detection results exhibit high accuracy and can precisely determine the qualitative and quantitative aspects of the measured object. However, owing to stringent equipment requirements, this method is unsuitable for on-site detection [36].

Although the current UV-Vis spectroscopy offers high accuracy for both qualitative and quantitative analyses, it has several limitations. Pretreatment of samples is often demanding, the equipment is expensive and cumbersome, and the detection process itself is relatively complex, making it unsuitable for portable and rapid on-site detection [37]. Additionally, in food analysis, some target substances exhibit weak UV absorption responses, and the complexity of real sample matrices further complicates accurate detection through UV-Vis spectroscopy [38]. Consequently, there is a pressing need to amplify the UV response signal of target substances, develop UV-Vis spectroscopy signal enhancement strategies, and simplify the spectrometer design to make the equipment portable and user-friendly [39].

Food quality analysis typically requires the detection of multiple indicators, which increases the complexity and number of detection steps. To address this, Yang [39] developed predictive models for fat, protein, lactose, and total solid concentrations in skimmed and high-pressure homogenized milk, using a partial least squares regression algorithm. The model could comprehensively detect the concentrations of fat, protein, lactose, and other substances in milk, and for daily on-site monitoring purposes, it could directly measure milk samples. The measurement time for milk samples was approximately 10 s, and no toxic substances were required to pretreat the milk samples. The portable milk quality detection system has the advantages of low cost, small size, rapid measurement speed, simultaneous detection, and convenient on-site operation. Regarding the detection of specific substances, Zhang [40] designed a probe based on gold nanoparticles to detect benzene-triacetic acid (BA) in the tremella, which enhanced the visibility of color changes under ultraviolet and visible light. Furthermore, when combined with a smartphone, this method overcomes the limitations of other techniques that require expensive instruments, professional analysts, time-consuming processes, and fixed detection locations, thereby achieving on-site, real-time, and rapid BA detection.

### 2.3. Surface-Enhanced Raman Spectroscopy

Surface-enhanced Raman scattering (SERS) is widely regarded as a powerful technique for detecting ultralow analyte concentrations. Its primary mechanism involves the generation of strong localized electric fields at plasma “hot spots,” such as sharp edges or nanoscale gaps in metallic structures [41]. SERS offers high sensitivity and the ability to produce molecular fingerprint information, making it suitable for real-time non-destructive field detection [42]. To amplify the analyte signal further, the development of specific and efficient SERS sensors is essential, which largely depends on the design of the SERS substrate. Currently, substrate materials for SERS sensors are broadly categorized into rigid and flexible types [43].

The substrates of traditional SERS sensors are generally composed of rigid materials, and methods such as the surface loading of mesoporous silica [44] and double-shell structure [45] are employed to enhance the Raman signal. Rigid material substrates have been applied in the food industry for a long time, resulting in more mature technology and a broader range of detectable objects. Flexible substrates utilize materials, such as nonwoven fabrics [46] and filter paper [47]. Compared with rigid materials, flexible materials can be cut into suitable shapes and conform to the surface of the object to be measured through wiping, wrapping, and other methods, making them more appropriate for on-site detection.

However, because of the low concentration of certain substances detected in food, the SERS signal is weak, limiting the application of SERS in field detection. Furthermore, most SERS detections utilize a rigid base, such as a silicon wafer or glass plate, which, when applied in field detection, imposes requirements on the properties and materials of the measured objects and is not suitable for widespread application [48]. Consequently, researchers have investigated nanomaterials with varying particle sizes and light detection methods from different perspectives to address this issue. Appropriate sizes of AuNPs can enhance the intensity of Raman signals, and suitable light methods can mitigate the influence of droplet size and properties on Raman signals [44]. However, owing to the necessity for material preprocessing, the preparation time is excessively long, rendering it unsuitable for field inspection applications.

The integration of SERS and molecular diagnostics enhances the sensitivity of SERS detection. Zhang [49] designed a SERS platform based on the CRISPR strategy for the sensitive detection of Aflatoxin B1 (AFB1), achieving a detection limit of 3.55 pg/mL. The original SERS signal was augmented to improve the specificity of SERS detection, thereby enabling the detection of low-concentration analytes. Through the incorporation of immunological techniques, the capture efficiency of target molecules was improved, the sensitivity of SERS detection was enhanced, and the pretreatment time was reduced. These advancements have enhanced the specificity, stability, and reliability of the SERS results. Najaf [50] established a method for the capture and detection of *E. coli* O157 in liquid media, such as apple juice, by combining nano-immunomagnetic separation (NIMS) and SERS. By immobilizing the trapping antibody for *E. coli* isolation and concentration on nanoparticles, they successfully reduced the total analysis time to less than one hour, with a minimum detectable concentration of 102 CFU/mL in apple juice. It could accurately detect bacilli at ultra-low concentrations within a short period, thereby reducing the risk of food poisoning. The integration of immunological techniques with SERS detection technology has shortened bacterial enrichment time. This approach is more suitable for field detection applications that require rapid results and short processing times.

### 2.4. Colorimetric Method

Colorimetric methods rely on the assessment of the color of a solution, either in its original state or after the addition of reagents, to estimate the concentration of the target analyte. This was achieved by comparing the color intensity of the solution to a standard using either the naked eye or a colorimeter [51]. Although simpler and more intuitive than other detection methods, colorimetric methods are limited by their sensitivity to light source variations, resulting in lower accuracy, sensitivity, and a narrower application range than techniques such as UV-VIS spectroscopy [52]. Despite these drawbacks, the colorimetric method is advantageous for food detection because of its ease of use and visual clarity. To maximize its potential, efforts should focus on enhancing the accuracy and expanding the application range of comparative colorimetry [53]. Recent advancements, such as the development of nanoparticles or immune antigen-based sensors, have improved the specificity and color intensity of colorimetric techniques, making them more reliable [54].

The traditional colorimetric method primarily depends on the color changes of dyes or solutions and offers a convenient, fast, and low-cost option. However, the color differences can be subtle, making them distinguishable with the naked eye and limiting the precision of the analysis [55]. Recently, with the rapid advancement of nanotechnology, nanases have emerged as highly suitable and specific sensor substrates for colorimetric detection [28]. These “nanozymes” feature specific binding sites, offering greater sensitivity and selectivity toward the target analyte. Despite these advantages, nanozyme designs often respond to a single signal, which can make the results vulnerable to external interference, thus reducing accuracy [56].

In the food testing process, the majority of testing standards pertain to concentration. Consequently, during field testing procedures, it is essential not only to detect the presence of a substance but also to accurately analyze its concentration. However, traditional fluorescence detection methods rely on visual assessment, which does not satisfy the requirements for precise on-site detection [57]. Furthermore, because colorimetry quantifies the results produced by a photoelectric colorimeter, the test outcomes may be influenced by the intensity of the light source under composite light irradiation, potentially affecting the accuracy of the results [35].

To address this issue, semiconductor particles can be combined to mitigate the interference of background fluorescence and enhance detection accuracy. Ulah [58] synthesized molybdenum disulfide and cobalt titanate nanocomposites and detected hydrogen peroxide using a colorimetric method on a filter paper platform. By integrating nanoenzymes and semiconductor particles, the stability of the sensor substrate was increased, the detection limit was 0.01 µM, which was far below the concentration range of hydrogen peroxide in drinking water set by the US Environmental Protection Agency (EPA), and the platform exhibited sufficient selectivity to potential interferences in biological samples. To improve the precision of the detection results, a signal amplification strategy was employed, combining nanoenzymes with the hybridization chain reaction, and a plasma colorimetric sensor was designed to enhance detection sensitivity. Zhou [59] incorporated secondary hybridization chain reaction (HCR) to determine chloramphenicol in honey. Through the specific binding of single DNA strands, the aptamer sensor significantly improved the specificity of the colorimetric reaction while simultaneously enhancing the signal strength owing to the initiation of secondary HCR, resulting in a more rapid and stable detection method.

### 2.5. Mid-Infrared Spectroscopy

Infrared detection is used to elucidate the molecular structure and properties of substances by analyzing the absorption spectra resulting from the differential absorption characteristics of infrared light. This technique enables the detection of multiple variables within the substance under examination, and the quantitative analysis of these variables can be conducted through model establishment [60]. Infrared detection typically involves placing the object of interest using an infrared spectrometer, subjecting the sample to infrared radiation, analyzing the infrared light absorption capacity of the sample, processing the obtained infrared spectrum, constructing a model, conducting spectral analysis, and subsequently analyzing the composition and structure of the object under investigation.

Food detection involves complex ingredients, which necessitate comprehensive consideration. In this context, infrared spectroscopy offers advantages such as simple testing procedures, rapid analysis speed, and low cost without pollution [61]. The spectra generated from the test results can be analyzed using algorithms, enabling the construction of models for a comprehensive analysis of the tested samples [62].

However, the results of infrared analysis are susceptible to interference from noise and varying background environments, necessitating preprocessing of the detection results and data accumulation to refine the algorithm and obtain accurate results [63]. Additionally, factors such as the position and angle of light incidence, as well as the distance between the sample and the light source, can introduce errors into the analysis [64]. In practical applications, low concentrations of certain nutrients in food produce weak infrared signals, leading to overlapping absorption bands. This overlap complicates the subsequent data analysis of the results and reduces the sensitivity of the infrared detection process.

To fully utilize the advantages of infrared detection, the results are typically combined with multivariate statistical analysis methods, considering multiple factors comprehensively to make more judgments by assessing the properties of the measured objects [65]. For example, Genangeli [66] conducted non-invasive detection of apple mildew cores using near-infrared spectroscopy. By employing two binary classification models, artificial neural network pattern recognition, and a Bagging Classifier with a decision tree, they developed a system for the non-destructive detection of mold in apple cores based on spectral analysis. In predictive tests, the first round achieved a 100% accuracy rate, which is particularly effective in detecting early-stage infections, whereas the second round achieved 97.15% accuracy, demonstrating the system’s ability for early mildew core detection. The system also improves the spectral range and overcomes the limitations related to the optical path length, thereby broadening the application scope of infrared spectroscopy. Similarly, Zhou [67] evaluated shrimp freshness by considering factors closely associated with freshness, such as protein content, chitin, and calcite peaks. Using mid-infrared optical fiber transient wave spectroscopy, which offers a longer optical range and higher sensitivity, combined with a partial least squares discriminant analysis model, achieved recognition rates of 87.27% and 90.28% for shrimp freshness, respectively. This method has proven to be a feasible, non-destructive, and in situ detection technique for assessing shrimp freshness. This also demonstrates the potential for rapid on-site infrared detection applications in the field.

## 3. Applications for On-Site Food Detection

### 3.1. Harmful Residues in Fruit and Vegetables

During the cultivation and processing of fruits and vegetables, these products are highly susceptible to contamination by microorganisms and their metabolites, which pose a significant risk to human health. Among these contaminants, rice yeast acid is a deadly toxin commonly found in rice flour, tremella mushrooms, and deteriorated grains, and its content is strictly limited. According to China’s national food safety standard, the permissible concentration of rice yeast acid in edible fungi should not exceed 0.25 mg/kg [68]. Zhang [40] developed a detection method utilizing the electrostatic interaction between the carboxyl group of rice yeast acid and gold nanoparticles, inducing nanoparticle aggregation and causing the color of the solution to shift from Burgundy to bluish purple. Within a certain concentration range, the absorbance ratio at 650 nm and 524 nm exhibited a linear increase, with a detection limit of 3.43 nM. This LOD was much lower than the national standards. Currently, this method has been experimentally validated on smartphones, and it meets the detection requirements.

Processed fruits and vegetables, such as vegetable salads, are susceptible to large-scale food poisoning incidents due to their exposure to high-traffic areas. When individuals or food items are contaminated with bacteria such as *E. coli*, the risk of widespread foodborne illness increases. Shigella, a pathogenic variant of *E. coli* that causes severe diarrhea and dysentery, has evolved antibiotic resistance, which complicates treatment options [69]. Feng [70] immobilized aptamers with strong affinity to Shigella flexneri on the surface of gold nanoparticles. In the presence of the target bacteria, the AuNPs aggregated, resulting in a color change in the solution. The absorbance ratio demonstrated a linear relationship with bacterial concentration. In the testing of real samples in the laboratory, the entire test could be completed within 20 min, indicating its potential as a diagnostic tool.

To protect fruits and vegetables from pests and diseases and to minimize the risk of contamination, pesticides are commonly applied during their growth and preservation. However, the application of pesticides may result in residues that pose potential health risks to consumers [71]. Several rapid detection methods have been developed and reported in the literature. For example, cyfluoxate, a pesticide sprayed on leaf surfaces, has been studied for its residues in agricultural products. Wu [72] developed a specific immunochromatographic strip based on the monoclonal antibody against cyfluoxate, enabling rapid and accurate detection of cyfluoxate on the surface of corn and brown rice. The detection limit for the T-line elimination was 100 μg/kg. In accordance with China’s maximum residue limits (MRLs) for pesticides in food, the maximum residued limit in brown rice is 0.1 mg/kg. The limit of detection (LOD) was lower than this residue limit. Currently, corresponding test strips have been developed, which could be well utilized in practical scenarios.

To improve the quantitative detection accuracy of pesticide residues, Liu [28] synthesized a metal–organic framework nanase NH_2_-CuBDC, which functioned as both a bionic catalyst and fluorescence tracer, enabling the colorimetric and fluorescence dual detection of organophosphorus pesticides. The sensor exhibited exceptional sensitivity, with detection limits (LOD, S/N = 3) of 1.57 ng/mL and 2.33 ng/mL in colorimetric and fluorescence modes, respectively. Even at low pesticide concentrations, the probe could still capture the pesticide, thereby fulfilling the purpose of screening.

In the experimental setup, the surface of the test object exhibited non-uniform characteristics. To address this complex real-world scenario, Lv [73] employed SERS detection using a novel durian-like Fe_3_O_4_@Au@Ag@Au (DFAAA) multilayer core–shell composite material as the substrate. This substrate demonstrated enhanced surface area and improved roughness, rendering it more suitable for diverse surfaces. This approach facilitates rapid and versatile detection of pesticide residues on different food surfaces, including fish and apples. The detection limits for malachite green and thiamine on fish and apple surfaces were determined to be 0.13 and 0.18 ng/cm^2^, respectively. It ensured that pesticides could still be detected under extremely low residual levels, thereby reducing the risk of poisoning.

In real-life scenarios, a single object is often contaminated by multiple pollutants. To enable the simultaneous detection of multiple pesticides, Ma [74] synthesized bimetallic core–shell Au@Ag nanoparticles as SERS substrates. This substrate enhanced the SERS signal intensity, facilitating the detection of mixed pesticides. A line diagram was generated based on the relationship between the Raman characteristic peak intensity and pesticide concentration, and a calibration curve correlating SERS intensity with pesticide concentration was established. The detection limits for acetamidine and thianidin in apple juice were determined to be 1.22 μM and 0.076 μM, respectively. It had been applied in real samples and could be detected at ultra-low concentrations, thereby minimizing the risk of food poisoning.

### 3.2. Harmful Residues in Animal Farming

During processing, storage, and transportation, meat and aquatic products are susceptible to microorganism contamination. Klebsiella pneumoniae (KP) and Acinetobacter baumannii (AB), primary causative agents of pneumonia, have been detected in the environment, and more recently, in food [75]. To mitigate the risk of pneumonia from consumption of contaminated food, Effah [76] employed vancomycin-modified nanomaterials as probes to selectively enrich and capture these bacteria. SERS was used to detect the target bacteria with a detection limit of 10 cells/mL. It enabled detection in environments with low bacterial load, which facilitated subsequent screening and control.

Following the cessation of respiratory processes in meat and aquatic products, these commodities become susceptible to microbial contamination during transportation and storage. Bacterial decomposition of proteins in meat or aquatic products results in the production of amines, leading to food deterioration. These amine compounds not only generate unpleasant odors in food but also pose potential health risks, including symptoms such as dizziness and nausea [77]. Mi [78] achieved sensitive detection of amine vapor based on photoinduced electron transfer from amine to excited PDI, with detection limits as low as 1.2 ppb, which was far lower than the regulatory limits for amines in food set by various countries, ranging from 50 to 100 ppm.

To prevent and treat epidemics in livestock, certain antibiotic drugs are frequently used during animal husbandry; however, these drugs may leave residues in the meat or dairy products. Amantadine, an anti-avian influenza drug, can produce residues in meat products that can induce dizziness and potential hallucinations in humans [79]. Pan [80] optimized background fluorescence quenching immunochromatography (bFQICA) and time-resolved fluorescence immunochromatography (TRFICA) to enhance the accuracy and sensitivity of amantadine detection. The detection limits of the two methods were 0.62 μg/kg and 0.29 μg/kg, respectively, which demonstrated superior performance compared to other conventional detection methods. To ensure detection even under conditions of extremely low residues, corresponding test strips have been developed.

Tetracycline is widely used in animal husbandry because of its potent antibacterial properties. However, excessive use can result in residue accumulation in animal products, which, when consumed by humans, can cause allergic reactions, gastrointestinal disorders, and other health issues [81]. To address this, Pan [82] developed a portable detection system for tetracycline residues in animal-derived foods. The system used carbon dots to provide stable fluorescence within photonic crystal hydrogels, whereas tetracycline was detected using molecularly imprinted polymers. This device enabled field detection with a detection limit of 0.067 μg/mL, and it still exhibited a distinct response under conditions of trace amounts.

To enhance the simplicity of the detection platform, Song [83] developed a novel portable detection system that integrates smartphones with test strips. By synthesizing a new physically doped Gd_0.9_@Eu_0.1_ proportional fluorescence probe, the fluorescence transitions from blue to red were observed. The detection limit was determined to be 14 nm, which was far lower than the 200 μg/kg and 100 μg/kg specified in China’s national food safety standards and EU food safety standards, respectively, thus meeting the detection requirements. However, during the detection process, data collection via a mobile phone still needed to be conducted under ultraviolet light, which limited its application in on-site detection and required further optimization.

Ampicillin, an antibiotic with potent bactericidal activity, is extensively utilized in medical practice. Incomplete absorption and metabolism of ampicillin can lead to its accumulation in animals or contamination of soil and water through animal feces. Subsequently, humans may accumulate ampicillin in their bodies after consuming contaminated food, potentially resulting in disruption of intestinal flora [84]. Chen [85] developed a detection method based on the fluorescence resonance transfer principle, employing upconversion particles (UCNPs) as energy donors and gold nanoparticles (AuNPs) as energy acceptors. Upon the addition of ampicillin, the initially quenched fluorescence was restored, and a linear relationship was observed between ampicillin concentration and fluorescence intensity. This method achieved a detection limit (LOD) of 3.9 ng/mL, which was lower than the maximum residue limits specified by the EU for milk and animal meat, being 4 μg/L and 50 μg/L.

### 3.3. Harmful Residues in Grain and Oil

Grains and oils are integral components of human nutrition, and consequently, their food safety has garnered significant attention from various stakeholders. Improper storage methods or transportation processes, particularly in warm and humid environments, can lead to the contamination of peanuts, corn, soybeans, and other grain and oil crops with AFB1, a secondary metabolic product. AFB1 exhibits potent toxicity and can induce various acute diseases, including liver cancer. As a result, the International Agency for Research on Cancer has classified it as a primary carcinogen. Numerous countries, including the European Union, the United States, and China, have implemented stringent regulations regarding maximum permissible levels in food [86]. The Chinese Pharmacopoeia Commission had also set the maximum residue limits for AFB1 and total aflatoxins at 5 μg/kg and 10 μg/kg. Wei [87] developed a Raman and fluorescence dual-signal AFB1 aptamer sensor. By combining silica nanoparticles loaded with a fluorescence signal source and aptamers with gold nanoparticles loaded with a Raman signal molecule and an aptamer, the sensor addresses the issue of inaccurate results due to the influence of a single signal source. The detection limit was 0.094 pg/mL.

To enhance the detection efficiency and speed of AFB1, Kong [88] developed a multimodal nanocase biosensor (TI_3_C_2_@SSDNA) for peanut AFB1 detection. This biosensor was constructed using a fluorescein (FAM)-modified aptamer and Ti_3_C_2_ nanocase (Ti_3_C_2_ Nes), which mimicked peroxidase activity. In the presence of AFB1, the toxin binds to the aptamers, causing the separation of ssDNA from Ti_3_C_2_, which results in alterations in both the fluorescence and colorimetric signals. The integration of this model into a smartphone platform enabled rapid AFB1 detection through the auxiliary imaging capabilities of the device. The detection system was mainly composed of an Android smartphone and a series of optical accessories, the latter of which mainly consisted of a specific black housing, an LED-integrated light source, a battery, and a sample holding slot. The detection limits for AFB1 were 0.09 ng mL^−1^, 0.61 ng mL^−1^, and 0.96 ng mL^−1^, respectively. Both of the above-mentioned probes had limits of detection that were far lower than the maximum residue limits, thus meeting the requirements for on-site detection.

Fumonisin is a secondary metabolite produced by Fusarium species, of which fumonisin B1 (FB1) is the most toxic and can cause neurotoxicity and carcinogenesis in humans. Consequently, it has been classified as a Group 2 B carcinogen by the International Agency for Research on Cancer. To address this, Niu [89] developed a novel rapid color sensor for FB1 detection in grains. The method relies on FB1’s ability to inhibit the formation of superoxide anions between Ag_3_PO_4_ nanoparticles (NPs) and dissolved oxygen, thereby disrupting the semi-laccase activity of Ag_3_PO_4_NPs. This change in laccase simulation activity served as a sensing signal. By integrating the sensor with a smartphone, rapid colorimetric analysis of FB1 can be performed, achieving a detection limit of 1.73 μg/L. On-site, color recognition software on a smartphone was directly utilized; the software generated a standard colorimetric card, enabling on-site portable detection and meeting the requirements for rapid detection.

Pb ions progressively accumulate in rice, tea, and other agricultural grain products through contaminated water and soil. Trace quantities of Pb ions can cause severe damage to the human nervous and endocrine systems and significantly impair brain development in children [90]. Liu [91] synthesized a probe by assembling glutathione (GSH) and 4-aminobenzoic acid (4-MBA) on silver-coated gold nanorods (Au@Ag NRs). Pb^2+^ in the solution was chelated by the free carboxyl groups on the probe, resulting in an enhanced SERS signal. The detection limit was determined to be 0.021 μg/L, ensuring the detection of lead ions under extremely low residual levels.

Aluminum-containing additives have been extensively utilized; however, when aluminum is combined with proteins, it directly impairs the development of the reproductive and nervous systems. Currently, there is no clear regulation regarding food additives that do not contain aluminum. Jiang [92] employed a portable positive fluorescence system (PFFFS) to determine aluminum content in flour. Following experimentation, the most suitable stabilizer and protective agent for aluminum ions were identified, which significantly enhanced the reproducibility, stability, and reliability of the PFFFS method. The device consisted of two optical modules, two control modules, a signal detection and amplification module, an information processing and storage module, and a Bluetooth printer. With a total length that was no more than 20 cm, it facilitated portability.

### 3.4. Harmful Residues in Processed Food

In food processing, certain unethical businesses attempt to reduce costs by illegally adding extraneous substances to food products, not only diminishing the quality of food but also potentially endangering food safety. The price of beef is higher than that of other meats; therefore, the addition of other meats is often prohibited [93]. Saleem [94] utilized positive fluorescence spectra to obtain fluorescence spectra from pure and adulterated meat, analyzed spectral data using principal component analysis, the least squares method, and other techniques, and established a prediction model. This model can be used to analyze 10% adulterated chicken; however, more data and further tests are required for lower levels of adulteration.

In addition to its flavoring properties, ginger powder derived from ginger also possesses traditional Chinese medicinal functions, such as dispelling cold and enhancing immunity. Owing to its powdery form, it is challenging to detect other powdery adulterants. Yu [95] employed Fourier transform near-infrared spectroscopy to analyze common adulterated powders, conducted a spectral analysis of corn starch, wheat flour, and soybean starch, and subsequently analyzed the obtained spectral data using various algorithms. Through comparison, the classification accuracies of the random forest and gradient-enhanced algorithms were found to be the highest, with the accuracy of discriminating between genuine and adulterated products reaching 100%. This method enables rapid identification of potential adulteration in samples. Similarly, Daszykowski [96] conducted Fourier transform near-infrared spectroscopy analysis of mixtures with varying adulteration concentrations and developed a model using principal component analysis and partial least squares regression. The sensitivity of the adjusted model for the verified sample exceeded 99%; however, its specificity was low, necessitating further testing.

Chia seed oil has recently gained significant attention due to its potential health benefits, particularly its capacity to reduce the risk of cardiovascular and liver diseases. However, the high cost of chia seed oil has led to the widespread use of this expensive oil as a cheap alternative. Mburu [97] conducted a study on the adulteration of chia seed oil with sunflower, rapeseed, and corn oils utilizing various optical detection methods to obtain different types of spectral data, including Raman, fluorescence, and near-infrared spectra. Through analysis of these spectra using the least squares method, researchers were able to detect the quantity of adulterated oil. After training, the resulting model demonstrated classification accuracy, sensitivity, and specificity exceeding 90%.

Honey serves not only as a flavoring agent but also has potential health benefits for humans. In practical applications, honey is frequently adulterated with multiple substances, including syrup, acids, and water [98]. Raypah [99] employed visible light and near-infrared spectroscopy to detect mixtures of honey with adulterants, including distilled water, apple cider vinegar, and high-fructose syrup; however, significant changes in the visible peak were observed only when adulteration exceeded 50%. An optimal prediction model was developed using least squares analysis to examine adulterated honey, enabling the detection of alterations in the water substructure of honey resulting from adulteration.

In practical detection scenarios, certain unscrupulous merchants employ multiple adulteration methods to circumvent traditional detection techniques [100], and the majority of current detection methods are limited to identifying only adulterants. Li [101] used Raman spectroscopy to identify adulterated oils, in which various oils were mixed with sesame oil. To address the fluorescence interference in Raman spectroscopy, Li employed activated carbon to adsorb fluorescent substances in edible oil, obtained the Raman spectrum of adulterated oil, selected significant variables through orthogonal partial least squares discriminant analysis, and developed a multiple adulteration identification model. The validation results demonstrated that 40 types of sesame oil mixed with four categories of adulterated oils could be accurately identified, rendering this method suitable for field detection.

### 3.5. Optical Detection of Food Raw Materials

Before food processing, there were many ways in which raw materials could be damaged or contaminated. Urbanization and global warming led to increased variability in crop yields and supplies, which exerted direct and indirect impacts on food quality. These sporadic variations affected microbial ecology and the spread of unexpected plant diseases, all of which had an impact on the food chain [102]. Seeds served as the basis for the transmission of key plant genetic information from one generation to the next. Arano [103], focusing on the detection of seed germplasm, developed a non-destructive detection method combining chaos theory with laser technology. The detection results were accurate with little damage to seeds, providing a practical framework for studying the dynamics of seed germplasm.

Water was a crucial raw material in food production and processing; therefore, ensuring the safety of the water used was of great importance. Recently, nanoplastics have been detected in various water bodies, including oceans, rivers, and bottled water. Thus, detecting nanoplastics in water was of significant importance. Qiu [104] used fiber-optic SERS probes for in situ detection of nanoplastics in water, achieving high SERS detection sensitivity and excellent spectral reproducibility. For 100 nm and 30 nm polystyrene nanoplastics in deionized water, the limits of detection were 0.32 μg/mL and 1.68 μg/mL, respectively. This was a further reduction compared to the previous limit of detection of 20 μg/mL, reducing the risk of using unsafe water.

The content of heavy metals in water bodies was also one of the important factors causing food risks. Due to their toxicity, heavy metal content in water bodies posed a serious threat to human health; therefore, developing functional materials suitable for metal ion detection and adsorption performance was very important. Cabrera [105] used edible gelatin as a probe and, utilizing the absorbance and luminescent properties of gelatin, established a rapid and simple method for detecting heavy metal ions in water. The probe exhibited high affinity for Fe^3+^ ions, with a limit of detection of 0.14 mg/L, meeting the permissible concentration of Fe^3+^ ions in drinking water specified by the WHO.

Milk was rich in nutrients and also served as a raw material for many dairy derivatives; therefore, it was consumed daily by billions of people of all ages worldwide, including millions of infants and children. In the production process of milk, determining the composition of raw milk was crucial. Nuzzi [106] developed an innovative technology for classifying raw milk based on the integration of speckle pattern imaging and artificial intelligence. Using algorithmic models to analyze the collected images, the best machine learning and deep learning models achieved an accuracy of 95%, providing a feasible method for the classification of raw milk.

As a food, fish was an excellent source of essential amino acids, unsaturated fatty acids, docosahexaenoic acid, and fat-soluble vitamins. Raw fish was usually preferred to preserve these nutrients and ensure optimal intake. However, fish meat tended to degrade rapidly, which provided suitable conditions for the reproduction of bacteria and/or parasites, increasing the risk of foodborne diseases. Miyazaki [107] measured the spontaneous fluorescence spectra of three different species of fish fillets under four excitation wavelengths and found that the spectral shapes and temporal changes varied among different fish species. Spectral analysis, using principal component analysis and curve-fitting, showed significant differences between different species. These findings provided a new approach for analyzing the different freshness levels of various fish species, making it suitable for subsequent development into a new optical on-site detection method.

## 4. Discussion

**Figure 2:**
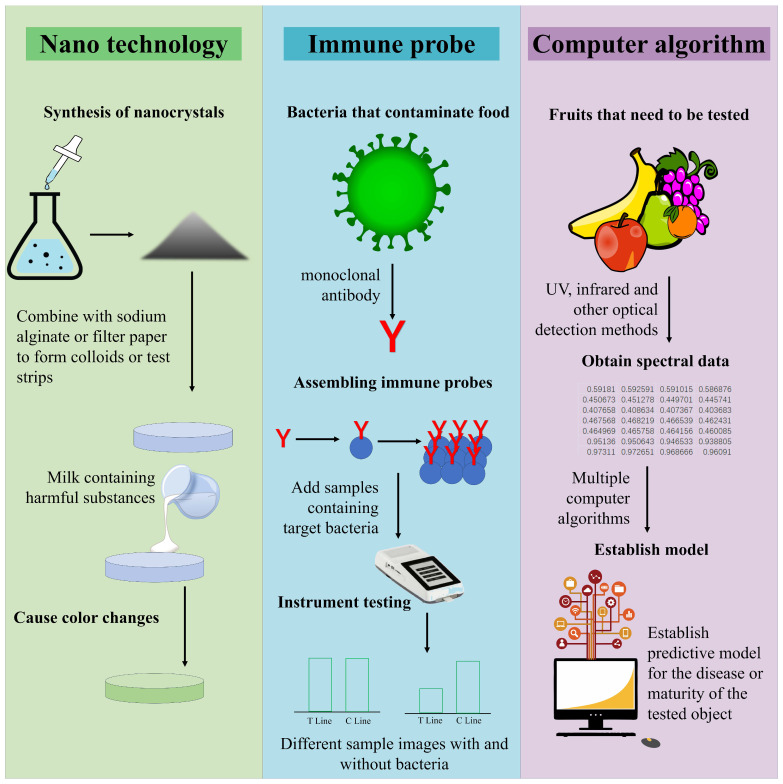
The combination of optical detection and other technologies.

**Figure 3:**
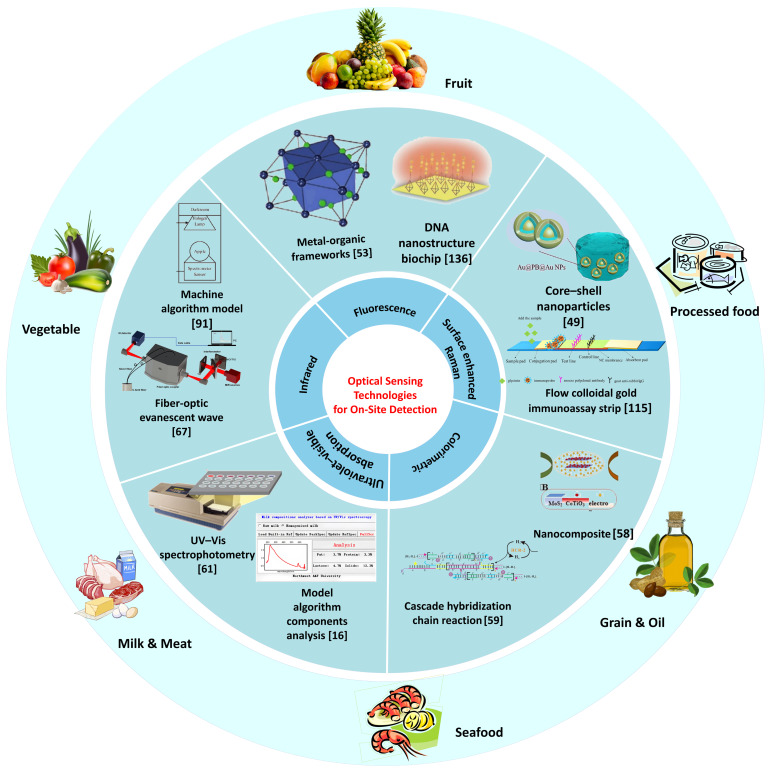
Application of optical detection in food inspection. Adapted with permission from Ref. [53]. Copyright 2024, Elsevier B.V. Adapted with permission from Ref. [132] Copyright 2024, Elsevier B.V.; Adapted with permission from Ref. [67]. Copyright 2023, Elsevier B.V.; Adapted with permission from Ref. [40]. Copyright 2022, Elsevier B.V.; Adapted with permission from Ref. [39]. Copyright 2022, Elsevier B.V.; Adapted with permission from Ref. [59]. Copyright 2022, Elsevier B.V.; Adapted with permission from Ref. [58]. Copyright 2023, Elsevier B.V.; Adapted with permission from Ref. [115]. Copyright 2023, Elsevier B.V.; Adapted with permission from Ref. [49]. Copyright 2023, Elsevier B.V.; Adapted with permission from Ref. [111]. Copyright 2024, Elsevier B.V.

**Figure 4:**
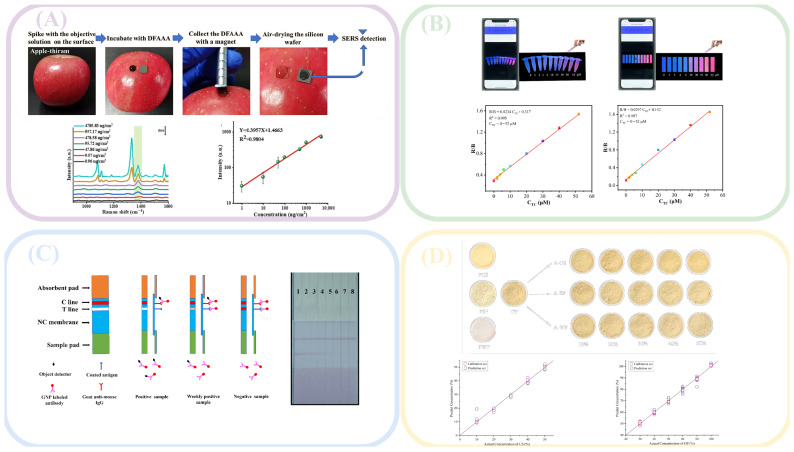
Various food non-destructive testing based on optical testing: (**A**) detection of pesticide on apple surface. Adapted with permission from Ref. [73]. Copyright 2024, Elsevier B.V.; (**B**) detection of tetracycline in real pork samples. Adapted with permission from Ref. [83]. Copyright 2023, Elsevier B.V.; (**C**) detection of cyhalofop-butyl in corn and brown rice samples. Adapted with permission from Ref. [72]. Copyright 2023, Elsevier B.V.; (**D**) detection of adulteration in powder of ginger. Adapted with permission from Ref. [95]. Copyright 2022, Elsevier B.V.

Optical detection had many advantages in on-site food inspection, whether it was the detection method of collecting light signals of the tested substance or the speed of obtaining detection results, as shown in Table 1, all of which demonstrated the potential for the development of optical detection in on-site inspection. Figure 1 shows the detection mode combining optical detection with new technologies. At present, optical detection applied to on-site food inspection could generally be divided into traditional optical signal conversion analysis mode and new material color change mode. The traditional optical signal conversion analysis mode mainly consisted of optical sensors and analysis instruments, and the current innovation was mainly focused on the development of new sensors and efficient analysis of optical signals. Combined with nanotechnology, organic metal frameworks, enzymes, and other substances, optical detection sensors had stronger aggregation power and better specificity for the analyte, making them more suitable for the micro-detection needs of food products. By combining different computer algorithms and optical detection analysis, based on the algorithm’s analytical and predictive capabilities, high-accuracy prediction of the analyte in food could be achieved. The color change mode generated by new materials was currently mainly focused on fluorescence detection and colorimetric detection. By combining probes with substrate materials, colorimetric or fluorescent reactions occur with the tested substance, producing color changes that could be collected and resolved by the naked eye or mobile phone, making it faster and more convenient. Therefore, as shown in Table 2, optical detection had various applications in on-site inspection. Figure 2 shows the different applications of optical detection in on-site inspection. Figure 3 shows the application scenarios of optical detection in actual food inspection. Figure 4 illustrates the development of optical inspection in non-destructive testing of food during the experimental process.

Here, we focus on discussing some of the challenges of optical detection in adapting to on-site food inspection, including the design of optical sensors, analysis of optical signals, and detection accuracy. Based on the current development status, we analyze and summarize the challenges of applying optical detection technology to on-site food inspection, hoping to provide reference directions for future research and development.

Regarding the optical detection probe, currently, in order to improve the specificity of the probe to the analyte, it is usually combined with nanoenzymes and immune technology, utilizing its characteristic of specific binding to the analyte at the molecular level, resulting in high detection accuracy and low detection limit. It is worth noting that the current molecular probes, such as nanoenzymes, are highly unstable and difficult to synthesize. At present, there are methods in the medical field to combine them with carbon point hydrogels to improve their usability and stability. However, the bacterial environment in the food field itself was more complex than that in medicine. How to ensure the specificity of molecular probes in field detection needs further exploration.

Regarding optical signal analysis, currently, the computer analysis mode that uses algorithms to denoise spectra and establish prediction models has developed well. By utilizing the fast computational speed and efficient information processing of computers, it is possible to quickly make highly accurate predictions of food status. Sahu [108] used a machine learning-supported spectrophotometer to perform non-destructive spoilage detection on sliced bread, and the classification accuracy reached over 95%. However, currently, there are no specific algorithms or trial rules for spectral processing. Most models mainly relied on early-stage experimenters to use enumeration methods to test the compatibility between algorithms and spectra, which resulted in heavy early-stage work and could not cover all algorithms. The optimal performance of the model cannot be guaranteed. At the same time, the establishment of the model mainly relied on the collection of preliminary data, by collecting spectral data of different states required for food and summarizing and debugging the prediction model. Guo [109], for different optical signals of various apple varieties, selected different algorithms for processing. The results showed that different algorithms performed differently in distinguishing between different categories of apples. Although appropriate algorithms achieved relatively high accuracy, properties such as color and pH possessed by the same apple still required multiple algorithms for determination. However, the damage, contamination, and illness caused by food are complex and varied and were greatly influenced by individual factors, environmental factors, and human factors. Even if a large amount of data was collected, it was difficult to generalize. Prediction models were often only applicable to food testing in specific situations, and their scope of use was very limited. While upgrading algorithms, more attention needed to be paid to how to adapt to individual differences in different foods in the development of predictive models.

**Table 1:** Advantages and disadvantages of combining optical detection with new technologies for on-site food detection.

Detection	Methods	Advantages	Disadvantages	Reference
Fluorescence	Nanozymes	Simple, highly selective, and sensitive	Potential cytotoxicity and food safety to be verified	[110]
	DNA probe	High stability and good universality in small molecule detection	The preparation process is complex, and the cost is higher compared to general food testing	[111]
UV-VIS Spectroscopy	Molecular absorption spectrophotometry	Easy to operate, high quantitative selectivity	The preprocessing method is complex	[112]
	Data processing algorithm	Comprehensive and accurate analysis, fast detection speed	Requires a large number of samples for calibration	[113]
Surface-Enhanced Raman	Genetic tools	Strong specificity and strong signal and high sensitivity, specificity, and accuracy	The substrate is expensive, subject to interference in complex environments	[114]
	Immune	Highly sensitive and specific	The synthesis process is complex; antigens are easily destroyed during food processing	[115]
Colorimetric	Nucleic acid amplification techniques	Easy to operate, low equipment requirements, and simple sensor design	Low accuracy and stability need improvement	[116]
	Nanozymes	Capable of detecting target molecules quickly, sensitively, and conveniently	There is relatively little research on the detailed mechanism; nonspecific adsorption affects the accuracy of the results	[116]
Infrared	Machine algorithm model	Comprehensive assessment without injury; economical and portable	Model interpretability needs to be developed	[117]
	Fourier transform infrared spectroscopy	Quick, no damage to the tested object, efficient and simple	Wide band with severe overlap between bands	[118]

Regarding the smartphone color signals, in optical detection methods, such as fluorescence and colorimetry, based on significant color changes, combined with the signal acquisition capability of smartphones, optical signals were converted into data information, making detection more convenient, rapid, and intuitive, which met the requirements for current on-site rapid detection of food. Wang [119] designed and synthesized a new probe for the sensitive and selective quantification of SO_2_ in food and environmental water. The experimental results showed that the probe produced a significant color change within 40 s; combined with the signal acquisition capability of smartphones, the quantitative detection range was broader. However, the current detection results were mainly judged through visual observation and mobile app judgment. During on-site testing, changes in environmental light sources, individual differences in testing personnel, and different models of mobile phone cameras could all lead to color judgment deviations, resulting in food detection errors. Hu [120], for the detection of nitrite, assembled a fluorescent and colorimetric dual-mode probe and developed a detection method based on smartphones and test strips. However, the test strips mainly relied on visual discrimination for concentration gradients, failing to yield highly accurate quantitative results. Moreover, to ensure the stability of the light source for photography, smartphones require the addition of a designated light source with a specific wavelength. This detection method was easily affected by environmental and individual differences, and the accuracy of detection is difficult to guarantee. Therefore, further exploration and solutions were needed to eliminate individual differences and environmental influences.

**Table 2:** Different applications of optical inspection in food inspection.

Analyte	Analyte	Optical Sensing Technology	Support Materials	Detected Data	Reference
fruit and vegetables	Maturity of kiwifruit	Infrared	Fourier transform infrared spectroscopy	R_p_^2^ 0.92	[121]
	Capsicum fruit defect	Infrared	Machine algorithm model	accuracy 98%	[122]
animal farming	Vibrio parahaemolyticus in cod	Fluorescence	DNA probe	LOD 39.00 CFU/mL	[123]
	Clenbuterol	SERS	Immune	LOD 0.48 pg/mL	[124]
	Milk powder adulteration	Infrared	Fourier transform infrared spectroscopy	LOD 0.25%	[125]
grain and oil	Adulteration of perilla oil	UV-VIS spectroscopy	Data processing algorithm	R_p_ 0.99	[126]
	Genetically modified soybean products	Fluorescence	DNA probe	LOD 0.20%	[127]
processed food	Wine-producing area	UV-VIS spectroscopy	Data processing algorithm	accuracy 100%	[128]
	Benzoates in beverages	Colorimetric	Nanozymes	LOD 0.33 μM	[129]
	Coffee acid produced by wine	Fluorescence	Nanozymes	LOD 18.90 nM	[130]
	Casein phosphopeptide	Colorimetric	Nanozymes	LOD 0.27 μg/mL	[131]

Note: LOD = limit of detection; Rp = prediction correlation coefficient.

Regarding optical detection equipment, currently, optical detection equipment mainly consists of probes, light sources, sample chambers, receivers, etc. The detection equipment ensures the stability and accuracy of the on-site detection results. Shin [133] achieved the classification of food quality grades and safety detection of soft materials through high-speed mechanical grating scanning imaging. However, components such as light sources and sample chambers in detection equipment greatly increased the cost of detection. Chen [134] used hyperspectral technology to perform comprehensive imaging of eggs; the defect detection accuracy of this method was 100%, with a defect detection time of 31 ms, achieving high accuracy and efficient detection in a laboratory environment. However, hyperspectral instruments were expensive, occupied a large space, and were not suitable for on-site detection applications. Food testing mainly faces daily life, and the value of the tested substances was usually not high. Overly expensive testing equipment was difficult to apply in practice. Therefore, how to reduce detection costs while ensuring detection accuracy was also a development direction that needs to be considered in the future.

**Figure 5:**
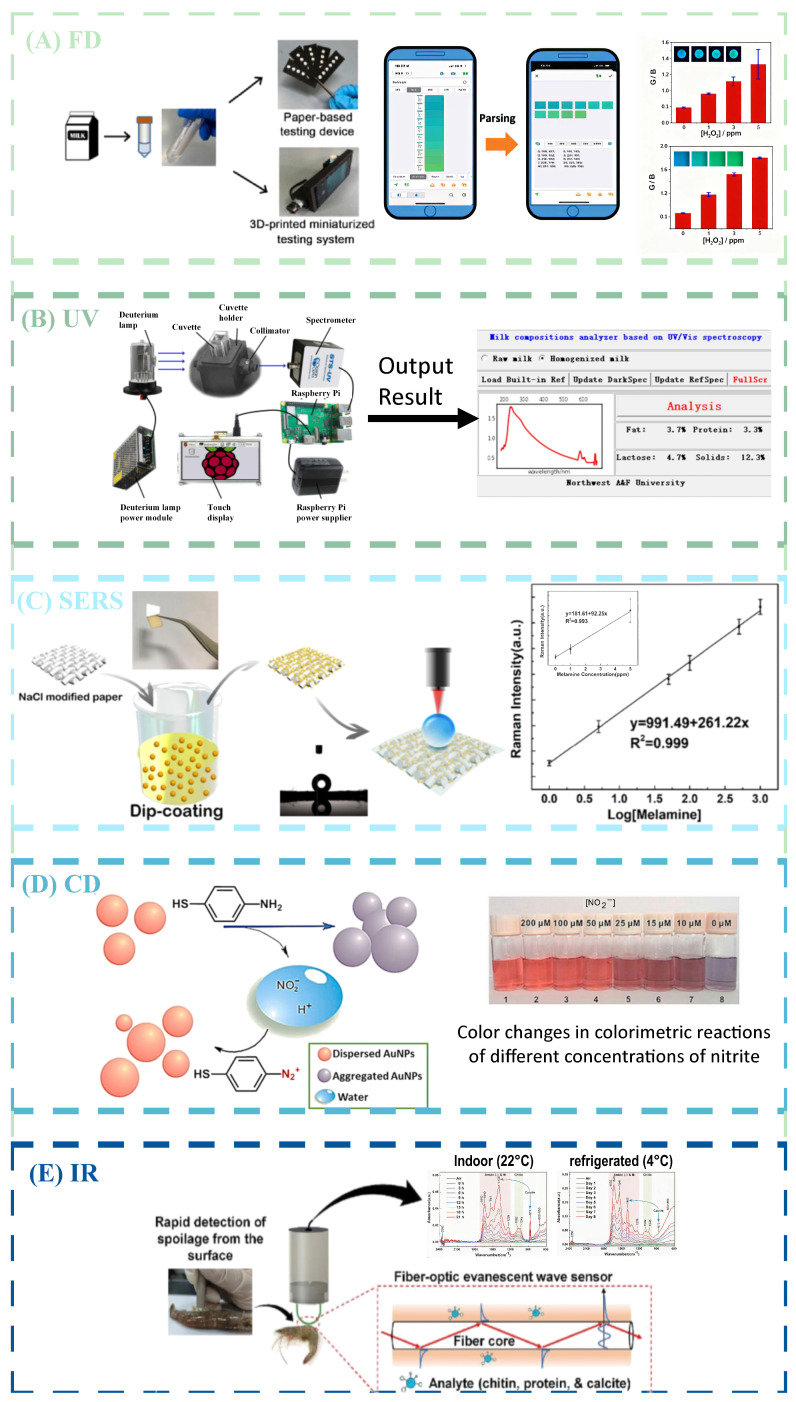
Non-destructive detection techniques based on imaging with optical inspection: (**A**) detection of hydrogen peroxide using fluorescence detection combined with smartphones. Adapted with permission from Ref. [136]. Copyright 2023, Elsevier B.V.; (**B**) detection of milk using ultraviolet–visible absorption spectroscopy. Adapted with permission from Ref. [39]. Copyright 2022, Elsevier B.V.; (**C**) detection of milk using SERS Adapted with permission from Ref. [47]. Copyright 2019, Elsevier B.V.; (**D**) detection of nitrite using colorimetric detection. Adapted with permission from Ref. [54]. Copyright 2021, Elsevier B.V.; (**E**) detection of shrimp freshness using mid-infrared fiber-optic evanescent wave spectroscopy. Adapted with permission from Ref. [67]. Copyright 2023, Elsevier B.V.

Regarding standardized validation and analysis, many advanced on-site food detection methods had already been applied and could maintain accurate detection results under complex and highly interfering on-site environments. Moreover, comparative analyses showed that their detection limits were all lower than the national regulatory standards, as shown in Table 3. Wang [119] designed and synthesized a novel probe for the sensitive and selective quantification of SO_2_ in food and environmental water. Furthermore, a portable TN-based visual test strip was developed to enable colorimetric/fluorescent dual-mode analysis for on-site SO_2_ quantification in different matrices. The detection range was 0–100 µM under ultraviolet light excitation, making it the first method to fully cover the range specified by the World Health Organization (63.16 µM). However, some experimental methods had only been validated in laboratories and had not yet been promoted for practical use. In particular, certain technologies overemphasized detection speed and convenience, resulting in reduced detection sensitivity that even failed to meet the requirements of national regulatory standards. Shi [135] developed a colorimetric and fluorescent probe capable of detecting high total polar materials (TPMs) in edible oil through a distinct blue color change. The probe could be applied to samples containing 26.9% TPM with good fitting performance. Despite its fast detection speed and obvious response, it did not comply with the TPM limits established by some countries—for example, Germany had set a mandatory TPM limit of 24%. Therefore, for the research and development of rapid on-site food detection technologies, validation should not only focus on speed and specificity but also, more importantly, on whether the detection limit meets the regulatory standards. Meanwhile, with the continuous advancement of research on food detection technologies, on-site applications have become increasingly widespread. On-site detection was popular due to its rapidity, but it was also susceptible to multiple interfering factors that affected the detection limit. Thus, corresponding standards for on-site detection should be formulated in the future to clarify the scope of application, usage scenarios, and operation methods of on-site food detection. Special emphasis should be placed on the positioning of on-site food detection—such as whether the results could be recognized as authoritative detection outcomes, whether they were only for preliminary screening, and whether further accurate detection was required afterward. Most importantly, appropriate limits for on-site food detection should be established to lay a foundation for the wider application of on-site food detection technologies. Figure 5 showed the non-destructive testing technology based on optical imaging

**Table 3:** Comparison of detection limits and national standards worldwide.

Analyte	Optical Sensing Technology	LOD	National Food Safety Standards	Corresponding Standards	Reference
hydrogen peroxide	Colorimetric	0.01 µM	25 to 50 ppm	US Environmental Protection Agency	[58]
rice yeast acid	UV-VIS spectroscopy	3.43 nM	0.25 mg/kg	China’s national food safety standard	[40]
cyfluoxate	Colorimetric	100 μg/kg	0.1 mg/kg	China’s maximum residue limits	[72]
amine vapors	Fluorescence	1.2 ppb	50 to 100 ppm	US Food and Drug Administration	[78]
tetracycline	Fluorescence	14 nM	100 μg/kg	EU food safety standards	[83]
ampicillin	Fluorescence	3.9 ng/mL	50 μg/L	EU food safety standards	[85]
AFB1	Raman	0.094 pg/mL	10 μg/kg	Chinese Pharmacopoeia Commission	[87]

Note: LOD = limit of detection.

## 5. Conclusions and Future Trends

Although traditional food detection methods employ bacterial culture colony counts, chemical tests, and other standard methods, they often require complex pretreatment procedures and yield slow results. Therefore, this study provides a detailed examination of the practical applications of various optical detection techniques for food analysis. At that time, the progress of optical detection technology mainly focused on the amplification and processing of optical signals. The amplification of optical signals mainly relied on the synthesis of new probes, including nanotechnology and enzymes. The processing of optical signals was mainly divided into intuitive color analysis and model-based processing of optical signals. The collection and processing of signals were combined with each other. A notable example is the integration of optical recognition with smartphone applications, which allows for rapid and convenient identification of detection strips. This innovation accelerated the development of optical detection technologies for food inspection. The integration and development of optical detection using nanotechnology, smartphones, and other technologies may present an effective approach for addressing these challenges, offering reliable sensitivity and expeditious detection processes.

Advancements in on-site food optical inspections represent a significant trend in food safety and quality control. But at that time, there were still limitations in the development of on-site detection. Given the complexity of real-world scenarios, it is often necessary to detect multiple types of contamination and adulteration in food products. Therefore, limiting the detection of individual substances is inadequate. Additionally, to minimize environmental interference during detection, nanomaterials or nanoenzymes have been incorporated into probes. Although many nanoparticles have been developed for optical inspection, their application remains largely confined to the laboratory, as scalable production methods for widespread applications have yet to be established.

Portable and rapid optical inspection technology holds great promise for advancing food field inspection. Owing to its simplicity and portability, this technology can be effectively utilized in various field inspection environments. The development of optical detection technology applicable to real-time field detection has been a consistent objective for researchers. The research focus at that time was on developing sensors with higher specificity. For the future, consideration could be given to strengthening the key step of extracting target substances from samples, laying the groundwork for more rapid and accurate analysis. Meanwhile, it was noteworthy that future research should prioritize the development and manufacture of multi-substance optical detection technologies, which, while maintaining sensitivity and performance, could analyze various pollutants or adulterants with a single detection result. Nanoprobes that had been developed by that time, emerging as components of optical detection methods, required attention to the safety of their use in future development. Additionally, future development should include portable testing devices, such as test strips, in addition to smartphone applications, to achieve comprehensive application in the source monitoring of food raw materials—such as agricultural products—and food safety. These advancements were of great significance to food safety and human health.

## Data Availability

No new data were created or analyzed in this study. Date sharing is not applicable.
